# Photoinduced Mass Transport in Azo-Polymers in 2D: Monte Carlo Study of Polarization Effects

**DOI:** 10.3390/ma13214724

**Published:** 2020-10-22

**Authors:** Grzegorz Pawlik, Antoni C. Mitus

**Affiliations:** Department of Theoretical Physics, Wroclaw University of Science and Technology, 50-370 Wroclaw, Poland; Antoni.Mitus@pwr.edu.pl

**Keywords:** photoinduced dynamics, azo-polymers, bond-fluctuation Monte Carlo, superdiffusion, light polarization

## Abstract

We studied the impact of light polarization on photoinduced dynamics of model azo-polymer chains in two dimensions, using bond-fluctuation Monte Carlo simulations. For two limiting models—sensitive to and independent of light polarization—their dynamics driven by photoisomerization of azo-dyes as well as by thermal effects was studied, including characterization of mass transport and chain reorientations. The corresponding schemes of light–matter interaction promote qualitatively different dynamics of photoinduced motion of azo-polymer chains. In particular, they can inhibit or trigger off a directed mass transport along a gradient of light illumination. The generic dynamics of single chains is superdiffusive and is promoted by breaking a symmetry present in the polarization independent model.

## 1. Introduction

### Photoinduced Mass Transport in Functionalized Azo-Polymers: Concepts

Thin films of azobenzene functionalized polymers [1] exposed to interfering polarized laser beams in degenerated two wave mixing (DTWM) experiment may develop periodic surface corrugation pattern called surface relief gratings (SRGs) [2,3]. The macroscopic mass transport at the surface of polymer thin film at temperature far below the polymer glass transition temperature $T_{g}$ is attributed to microscopic polymer chain movements which result from light-induced multiple trans↔cis photoisomerization cycles of azobenzene dyes attached to the chains. Nevertheless, its origin remains unclear and constitutes one of the challenges in polymer physics. A summary of this topic can be found in the review papers [4,5] and in [1]. The various general ideas and approaches include mean-field approach [6], pressure gradient scenario [7,8], photoexpansion and photocontraction effects [9], viscoelastic flow [10], inchworm-like motion [11], gradient force models [12,13], Navier–Stokes dynamics [14,15], random-walk approaches [16], stochastic models [17,18,19], light-induced softening [4,12], atomistic molecular dynamics simulations [20], and others.

The majority of theories are based on the concept of directional photofluidization or light-induced plasticization [4,12,21,22,23,24]. This scenario goes back to the seminal paper by [12], which introduces the concept of light-induced plasticization (motion of polymer chains) due to reorientation of chromophores (via photoisomerization cycles trans↔cis). On the contrary, an analysis of available experimental data [4,21,25,26] resulted in the conclusion [27] that there is no convincing experimental evidence of photofluidization. A competing theoretical approach [27,28,29,30], based on the scenario in which reorientation of chromophores (via trans↔cis cycles) and subsequent polymer chains movements can produce strong stresses in azo-polymer systems sufficient to generate lasting deformations of azo-polymers in glassy phase [31,32,33], provides a satisfactory explanation of behavior of azo-polymer systems interacting with light.

Those papers are oriented on the study of physical origin of the deformation which triggers off the motion of polymer chains. On the other hand, the characterization of the resulting dynamics of the chains is missing. Some attempts to study this aspect of photoinduced motion of azo-polymers are reported in [11,17,18,19] (see [34] for more details).

Some light was cast onto the corresponding dynamics by Monte Carlo (MC) bond fluctuation model [35] (BFM) of the photoinduced mass transport of azo-polymers, as proposed by our group [34]. The model, which mimics the effects of multiple photoisomerization cycles of functionalized dyes in a host polymer matrix in the presence of spatially inhomogeneous light illumination, satisfactorily explains experimental effects such as a directed mass transfer from bright to dark places and the fine structure of SRG-effect observed but either not discussed [36] or disregarded [37]. In particular, it reproduces the linear relation between the average velocity of centers of mass (CM) of polymer chains and gradient of the light illumination, postulated in macroscopic approaches [7,8,12,13]. Nevertheless, the “microscopic” origin of the photoinduced directed motion remains unclear, in part because of very limited knowledge about motion of single functionalized polymer chains illuminated with polarized laser light.

This topic was studied recently [38] and has revealed a complex MC dynamics of functionalized single chains at constant illumination as well as in the presence of its gradient, including subdiffusive, diffusive, and superdiffusive regimes. The study was done for chains in 3D and used a simplified version of transition probability trans→cis, in which the $\cos^{2}\theta$ term (see below) was approximated by a step function. As a result, nearly all azo-dyes underwent the photoisomerization transition independently on their actual orientation, rendering impossible an analysis of an impact of polarization of light on dynamics of the chains. This model, referred to as simplified polarization model (SPM), was also used to study the inscription of SRG [34].

The goal of this paper is to study the impact of polarization of light on the dynamics of single azo-dye chains in the two-dimensional (2D) case (chains are restricted to x−y plane) using a modification of the original 3D model [38]. The advantage of 2D case over its 3D counterpart is due to a reduction of a number of parameters necessary to characterize the dynamics as well as a simplicity of interpretation of the results. On the other hand, the 2D model correctly reproduces, in a qualitative way, some of the results of 3D system.

The classification of types of light induced motion of azo-polymers constitutes an important step towards establishing a sound “microscopic” physical picture of the corresponding dynamics, potentially offering a reliable starting point for simple mathematical models of physical phenomena in which photoinduced mass transport of azo-dye doped polymers plays the central role. For example, mathematical formalism of continuous time random walk [39] was used to model some aspects of directed mass transport of azo-polymers [38].

The current study belongs to a much wider class of research related to light use in the polymers field. Among the large variety of effects, let us mention developing of polymer-based materials by UV–Vis and gamma radiations and using UV cross linking [40,41,42], gamma sterilization [43], gamma functionalization [44], and surface modification [45]. Simulation methods developed in this paper can contribute to a deeper understanding of the underlying physical processes.

The paper is organized as follows. The next section introduces a 2D modification of 3D MC bond-fluctuation model of azo-polymers. MC dynamics of a reorientation of model azo-polymer chains is analyzed in Section 3.1, while the overall dynamics is characterized in Section 3.2, in both cases for constant linear polarization of light. The dynamics in the presence of an inhomogeneous light illumination is studied in Section 3.3. Finally, a discussion of the results is given in last section.

## 2. Materials and Methods

The methodology used in this paper has been described in detail previously [34,38]; below, we present its milestones.

### 2.1. Model of Azo-Polymer Chains in the Presence of Light Illumination

**Figure 1:**
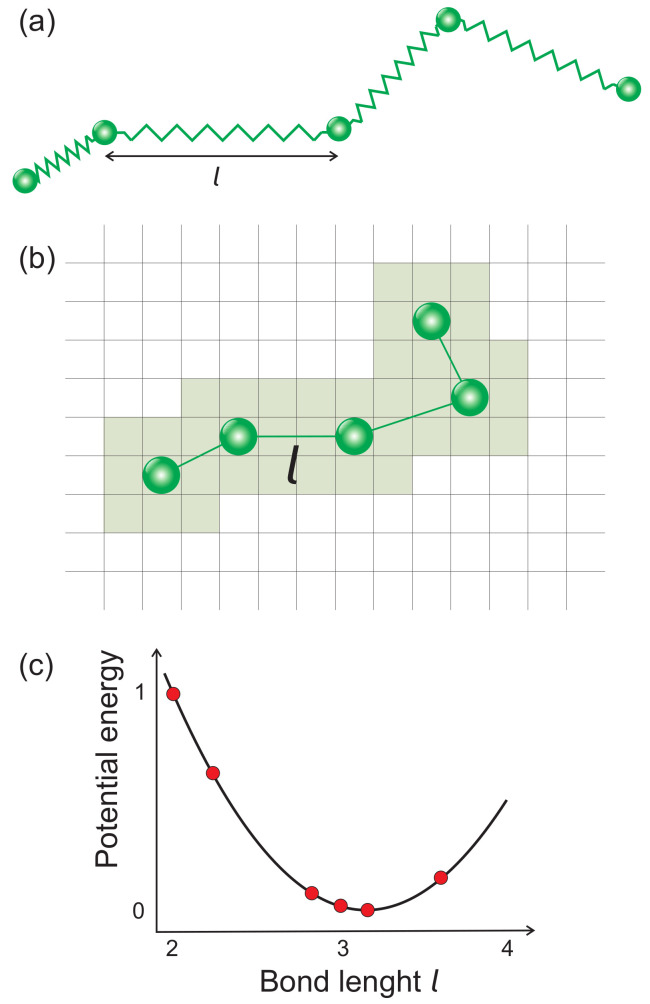
(**a**) Scheme of continuum bead-spring model; (**b**) lattice bond fluctuation model in 2D; and (**c**) potential energy of bonds in 2D BFM.

The polymer chains are simulated using Monte Carlo lattice bond-fluctuation model [35,46,47], which stems from continuum bead-spring model [48,49]. The monomers (Kuhn elements) occupy lattice sites; nearest-neighbor monomers along the backbone of the polymer chain are bonded by some potential (Figure 1). Due to the multitude of different bond lengths and bond angles, the BFM combines advantages of continuum and lattice models. The details are as follows.

A polymer chain in 2D [47] consists of *N* monomers which occupy the nodes of a square lattice. The bonds which join nearest monomers along the chain can take six non-equivalent orientations: bond lengths read (in lattice constants) 2, $\sqrt{5}$, $\sqrt{8}$, 3, $\sqrt{1}0$, and $\sqrt{1}3$. The corresponding bond stretching energies read (in units $E_{0}$ which sets the energy scale): 1.00, 0.64, 0.08, 0.02, 0.00, and 0.15 (Figure 1c).

**Figure 2:**
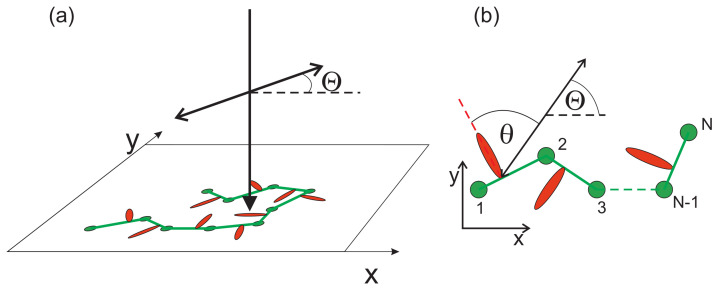
Geometry of the 2D azo-dye system (**a**). Polymer chains lie in x−y plane, light polarized in x−y-plane propagates along the *z*-direction (**b**).

The azo-dyes are attached close to one of the two monomers which form a bond; in trans state they are strictly perpendicular to the bond (see Figure 2). When illuminated with linearly polarized UV–Vis laser light, an azo-dye undergoes the transition trans→cis with probability *p* per unit of time (transition rate) [1]

(1) $$p\propto I_{L}\cos^{2}\theta ,$$

where θ stands for an angle between the long axis (transition moment) of trans molecule and direction of light polarization given by angle Θ. Figure 2 shows the corresponding geometry of the 2D azo-dye system for the light propagating along the *z*-direction and polarized in x−y-plane.

Transition rate *p* depends on an instantaneous orientation of the dye and, in general, varies along the chain (see Figure 2b). Thus, the model is sensitive to the polarization of light and is referred to as full polarization model (FPM). To develop a deeper intuition concerning the impact of light polarization on the MC dynamics of azo-chains, we introduce, in contrast, a model which is fully insensitive to the polarization, in which the transition probability *p* is constant and independent on an actual orientation of an azo-dye. The model is referred to as polarization independent model (PI model). The SPM used in 3D [34,38] is closer to PI than to FPM. Correspondingly, in this study, the transition rate *p* for a non-thermal movement reads:

(2) $$p=\left\{\begin{array}{cc} I(x,y), & \mathrm{polarization}\mathrm{independent}(\mathrm{PI})\mathrm{model}\\ I\left(x,y\right)\cos^{2}\theta , & \mathrm{full}\mathrm{polarization}\mathrm{model}(\mathrm{FPM}) \end{array}\right.$$

where 0≤I(x,y)≤1 denotes a dimensionless light intensity [38].

### 2.2. Monte Carlo Simulations of Functionalized Polymer Chains

The concept of Monte Carlo method comes down to using random numbers to estimate parameters of a probe sampled from a general population. In statistical physics, the widely used Metropolis Monte Carlo algorithm [50] was successfully implemented in polymer science [51,52]; other implementations are also used [53,54,55].

In general, Metropolis Monte Carlo algorithm [50] is used to sample a set of typical equilibrium configurations of a system of interacting atoms, liquid crystal molecules, polymer chains, etc. at absolute temperature *T*. To this end, one starts from some (*old*) configuration with energy $E_{old}$ and modifies it by performing trial movements of its constituent elements. Trial configuration generated in this way has energy $E_{trial}$, and it joins the set of configurations on the basis of Metropolis acceptance rule, which accepts it with probability equal to the smaller value of two expressions: 1 and $e^{-\left(E_{trial}-E_{old}\right)/\left(k_{B}T\right)}$, where $k_{B}$ denotes Boltzmann constant. If the trial configuration is rejected by acceptance rule, then *old* configuration becomes the new member of the set of configurations. This procedure is iterated, starting from the configuration newly added to the set. The thermodynamic parameters are monitored to estimate the onset of thermodynamic equilibrium. From now on, the same procedure samples a set of equilibrium configurations, used to evaluate averaged thermodynamic and structural parameters, as well as kinetic/dynamic effects.

In our simulations, the monomers performed two kinds of trial MC movements, namely thermal [47] and non-thermal, the latter due to the interaction of azo-dyes with light [34,38] discussed in the previous section. The former drives the system towards equilibrium, the latter out of it. The trial thermal movement of unit length was performed along randomly chosen lattice side. It was accepted if the following three conditions were met: (i) a length of a trial bond did not violate imposed restrictions; (ii) steric constraints were obeyed; and (iii) the Metropolis acceptance rule did not reject the movement [47].

Non-thermal MC movements mimic the effect on Newtonian mechanical forces and torques triggered by photoisomerization cycles trans↔cis in the presence of an optical field $I_{L}$ [34,38]. Namely, a photoisomerization transition of an azo-dye is accompanied by an additional, non-thermal trial movement (of unit length along one of the two directions x,y) of the monomer closest to the dye. The movement is accepted if Conditions (i) and (ii) formulated above are satisfied. Metropolis acceptance rule is not taken into consideration because this trial movement is not thermally driven—the typical energy of light quanta which trigger the photoisomerization transition (a few eV) is much larger than typical thermal energy at room temperature *T* ($k_{B}T\approx 3\times 10^{-2}$ eV).

The model does not account directly for the kinetics of cis→trans transitions: after the photoisomerization transitions, the molecules return to trans states.

One MC step (MCS) corresponds to a sweep of *N* trial movements of monomers, both thermal and non-thermal, and sets a unit of MC “time” *t* measured in the number of MCS. To avoid correlations in sampling the monomers, in both cases, the monomers were chosen in a random way.

The length of a typical run was $10^{4}$ MCS. This specific choice corresponds to an early phase of a directed mass transport in the process of inscription of SRG in 3D [34]. The simulations were done for N=25 at reduced temperature $T^{*}\equiv T/E_{0}=0.15$, slightly below the glass temperature $T_{g}^{*}\in \left(0.16-0.20\right)$ [47].

To improve the statistics of the data as well as for a purpose of a visualization, we studied an ensemble of $N_{0}=10^{3}$ independent polymer chains. In an initial configuration, the center of mass of each chain was localized in the center of 500×500 square lattice. The results were averaged over $N_{0}$ polymer chains. The corresponding experimental errors, represented by the standard deviations of the means, were in most cases small (see figure captions) due to the factor $\sqrt{N_{0}}$.

The MC simulation code was written by one of us (G.P.).

## 3. Results

### 3.1. Macromolecular Ordering of Azo-Polymer under Constant Linearly Polarized Light Illumination

Experimental results [56] indicate that exposure of azo-films to a linearly polarized light leads to a reorientation of azobenzene units perpendicularly to the polarization direction. This effect is related to the preferable reorientation of the chain along polarization direction.

**Figure 3:**
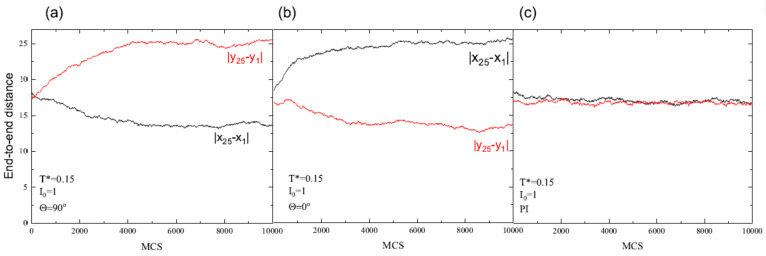
MC dynamics of averaged end-to-end distances Δx (red) and Δy (black): for FPM with $\Theta =90^{\circ }$ (**a**) and $\Theta =0^{\circ }$ (**b**); and for PI model (**c**). $I_{0}=1$. Experimental errors (not shown) are approximately 1.

In what follows, we study this process using the models introduced above. A simple measure of an average orientation of a system of chains is the difference of average end-to-end distances along *x* and *y* directions: Δ=|Δx−Δy|, where $\Delta x=|x_{25}-x_{1}|$, $\Delta y=|y_{25}-y_{1}|$. For an isotropic distribution of orientations of the chains, Δ=0. On the other hand, the inequality Δ≠0 stands for some degree of orientational order. Figure 3 shows the evolution of parameters Δx and Δy in MC-time for FPM with $\Theta =90^{\circ }$ (Figure 3a) and $\Theta =0^{\circ }$ (Figure 3b) and for PI model (Figure 3c). In FPM, the chains display a preferred orientation along the direction of light polarization. For example, for $\Theta =90^{\circ }$, Δx<Δy and, correspondingly, the chains are more stretched along the polarization direction (*y* axis) than along *x* axis. To avoid misunderstanding, we point out that the actual values of Δx and Δy correspond to a rather low degree of orientational order, because a system of perfectly oriented chains along, e.g., the *x* axis, is characterized by Δx∈(48,72). On the contrary, Δ=0 for PI model, which suggests that the orientations of the chains are random.

**Figure 4:**
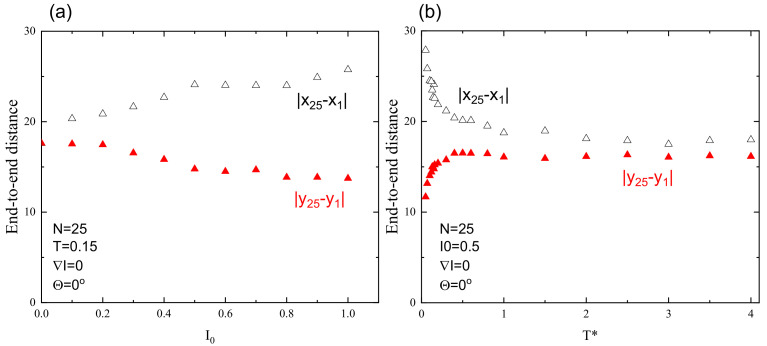
Averaged end-to-end distance after illumination time of $10^{4}$ MCS as function of: light intensity $I_{0}$ (**a**); and temperature $T^{*}$ (**b**) ($I_{0}=0.5$). In both cases, $\Theta =0^{\circ }$. Experimental errors are of the size of graphical symbols.

The degree of orientation, represented by parameter Δ, depends on light intensity and temperature (see Figure 4). Those results were obtained on the basis of the above plots (Figure 3) be performing linear regression analysis in last $10^{3}$ MCS to smooth out the fluctuations of the data. As expected, orientational order increases with increasing light intensity and decreases as the temperature approaches the glass temperature $T_{g}^{*}$. Nevertheless, well above $T_{g}^{*}$ the residual orientational order is present due to the fixed polarization direction; intensity $I_{0}$ plays a role similar to that of constant magnetic field for Ising model [57].

Another method of characterization of averaged orientation of the chains uses the concept of orientational order of polymer bonds adopted from the physics of liquid crystals, represented by the second rank symmetric and traceless tensor [58]:

$$\hat{Q}=\left(\begin{array}{cc} Q_{xx} & Q_{xy}\\ Q_{xy} & -Q_{xx} \end{array}\right).$$

The orientation of a single bond is described by a director $\hat{n}=\left(n_{x},n_{y}\right)$ [58]. In the coordinate system where $\Theta =0^{\circ }$ (Figure 2b), $\hat{n}=\left(\cos\theta ,\sin\theta \right)$ and tensor $\hat{Q}$ can be written in the component notation:

$$Q_{\alpha \beta }=\frac{1}{N_{B}}\sum_{i=1}^{N_{B}}\left(2n_{i,\alpha }n_{i,\beta }-\delta_{\alpha \beta }\right),\;\alpha ,\beta =x,y,$$

where $n_{i,\alpha }$ denotes the αth component of director $\hat{n}$ for *i*th bond, $N_{B}=N-1$ is the number of bonds, and $\delta_{\alpha \beta }$ is the Kronecker delta function. Tensor $Q_{\alpha \beta }$ characterizes the average orientation of the bonds as well as the degree of the orientational order, the latter in terms of a scalar order parameter S=λ, where ±λ are the eigenvalues of $\hat{Q}$. Parameter *S* takes values between 0 for a completely disordered phase and 1 for a completely ordered phase.

**Figure 5:**
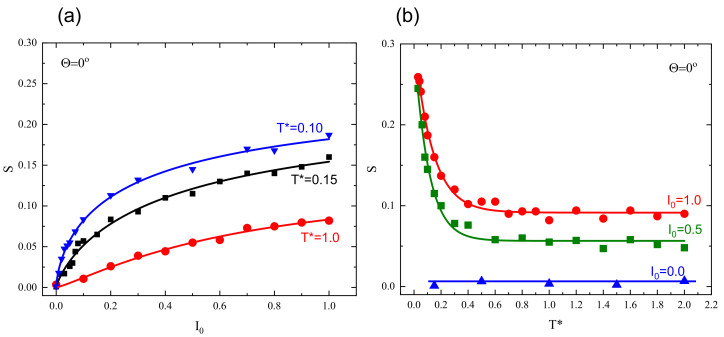
Scalar order parameter *S* for FPM after illumination time of $10^{4}$ MCS as function of: light intensity $I_{0}$ (**a**); and temperature $T^{*}$ (**b**). Experimental errors are of the size of graphical symbols.

Dependence of *S* on temperature and light intensity for FPM is presented in Figure 5. As expected, the orientational order increases with increasing illumination intensity $I_{0}$ and decreases as the temperature increases. In a close analogy with end-to-end characterization, the residual order remains well above glass temperature.

**Figure 6:**
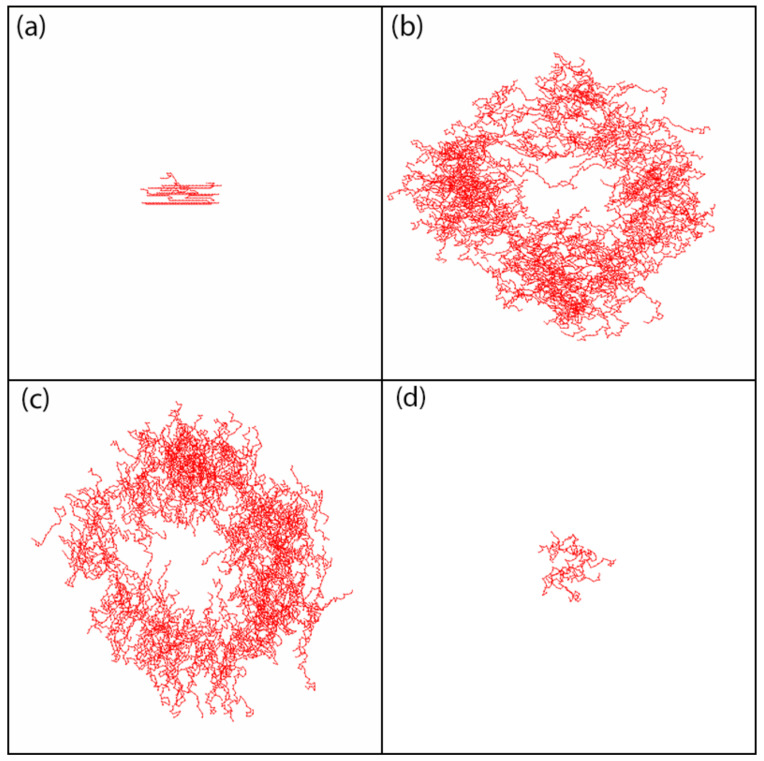
Configurations of chains after 6000 MCS, $I_{0}=1$: FPM without thermal movements, $\Theta =0^{\circ }$ (S=0.81, 10 chains are shown) (**a**); FPM with $\Theta =0^{\circ }$ (S=0.15, 300 chains are shown) (**b**); FPM with $\Theta =90^{\circ }$ (S=0.15, 300 chains are shown) (**c**); and PI model (S=0.002, 10 chains are shown) (**d**). Only a 300×300 part of the lattice is shown.

An overall orientational order of the bonds is the result of two competing processes: ordering process, due to photoisomerization cycles and disordering process, caused by thermal movements. Visualization of instantaneous configurations of the chains offers a much deeper insight into the photoinduced dynamics of the chains and the role played by both processes than solely on the basis of scalar order parameter *S*. Figure 6 shows those configurations after 6000 MCS in three important cases. Firstly, the FPM with the dynamics driven solely by photoisomerization events (thermal movements are not taken into account) promotes a high degree of orientational order: S=0.81 (Case (a)). Most of the chains lie along the polarization direction (*x* axis); no translational motion of the chains is found. The dynamics changes dramatically when the thermal movements are accounted for ($\Theta =0^{\circ }$ and $\Theta =90^{\circ }$, Cases (b) and (c), respectively). Namely, a strong transport of individual chains away from their initial positions is present, generating an empty area in the middle of the lattice. In both cases, a general tendency for the chains to order along the preferred polarization direction is present, but the thermal movements reduce the degree of orientational order: S=0.15. Finally, a third type of dynamics arises for PI model—there is no orientational order (S=0.002), but a limited transport of chains is present (Case (d)).

### 3.2. Macromolecular Displacement of Azo-Polymer under Linearly Polarized Light Illumination

#### 3.2.1. Superdiffusive MC Dynamics

**Figure 7:**
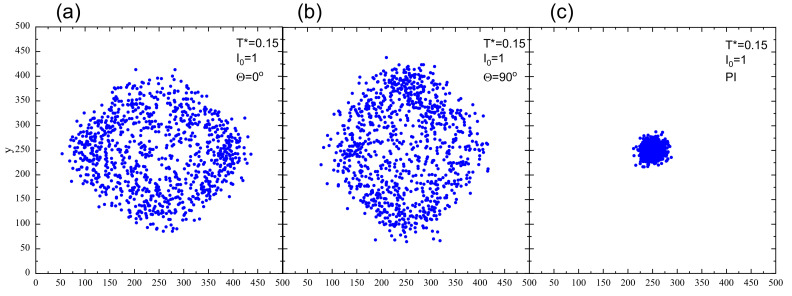
Final positions of CM of $10^{3}$ chains after $10^{4}$ MCS: linear polarization with $\Theta =0^{\circ }$ (**a**) and $\Theta =90^{\circ }$ (**b**); and PI model (**c**). $I_{0}=1$.

The presence of nearly empty centers in Figure 6b,c indicates that the dynamics is not diffusive. To characterize it, we use the method worked out in [38], which amounts to an analysis of the mean-square displacement of center of mass of azo-polymer polymer chains in function of number of MCS for a given pattern of light polarization. Initial position of CM of each chain lies in the center of the system ($x_{0}=250$, $y_{0}=250$). Figure 7 shows the final positions of all centers of mass after elapsed MC time $t=10^{4}$ MCS, in the case of linearly polarized light, for both models: FPM and PI. Qualitatively, the typical range of displacement of chains in the former case is much larger than in the latter. We conclude that the heterogeneity of photoisomerization cycles resulting from $\cos^{2}\theta$ term in Equation (2) promotes a much stronger dynamics than PI model. To avoid misunderstanding, we point out that the empty area around the starting point is not as clearly expressed as in Figure 6 because of a larger number of chains displayed in this case.

The mean-square displacement is calculated in the following way [38]. The vector ${\vec{r}}_{i}^{\left(CM\right)}\left(t\right)=\left(x_{i}^{\left(CM\right)}\left(t\right),y_{i}^{\left(CM\right)}\left(t\right)\right)$ of CM of a single *i*th chain is calculated after each MC step. The square of the displacement of CM from the initial position at t=0 reads ${\left(\Delta {\vec{r}}_{i}^{\left(CM\right)}\right)}^{2}\left(t\right)={\left({\vec{r}}_{i}^{\left(CM\right)}\left(t\right)-{\vec{r}}_{i}^{\left(CM\right)}\left(0\right)\right)}^{2}$. Because of fluctuations of this random variable, we averaged it over an ensemble of $N_{0}$ independent chains to obtain the average square ${\left(\Delta {\vec{r}}^{\left(CM\right)}\right)}^{2}\left(t\right)$ of the displacement of CM, which characterizes the random walk of a single chain:

(3) $${\left(\Delta {\vec{r}}^{\left(CM\right)}\right)}^{2}\left(t\right)=\frac{1}{N_{0}}\sum_{i=1}^{N_{0}}{\left(\Delta {\vec{r}}_{i}^{\left(CM\right)}\right)}^{2}\left(t\right).$$

In the case when the log-log plot of ${\left(\Delta {\vec{r}}^{\left(CM\right)}\right)}^{2}\left(t\right)$ becomes linear, a power law characterized by an exponent γ is present:

(4) $${\left(\Delta {\vec{r}}^{\left(CM\right)}\right)}^{2}\left(t\right)\propto t^{\gamma }.$$

Three different types of dynamics are classified depending on exponent γ: subdiffusive (γ<1), diffusive (γ=1), and superdiffusive (γ>1) [38].

**Figure 8:**
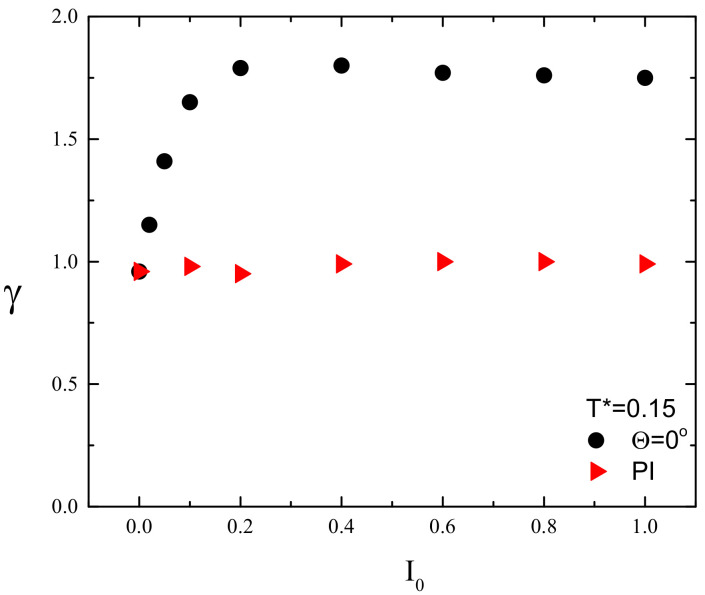
Plot of exponent $\gamma (I_{0})$ for FPM with linearly polarized light (black circles, $\Theta =0^{\circ }$) and for PI model (red triangles). Experimental errors are of the size of graphical symbols.

Figure 8 shows the plots of exponent γ in function of light intensity $I_{0}$ for FPM with linear polarization and for PI model. In the former case, the dynamics is superdiffusive (γ>1). In particular, for sufficiently high illumination, e.g. $I_{0}>0.2$, the value of exponent γ is around 1.8, which corresponds to nearly ballistic motion. This type of dynamics is responsible for empty regions in the center of the lattice (Figure 6b,c and Figure 7a,b). On the contrary, the PI model promotes standard diffusion with γ=1, responsible for characteristic spreading out the distribution of CM of chains in Figure 7c.

#### 3.2.2. Inhomogeneous Illumination: Continuous Polarization Rotation

**Figure 9:**
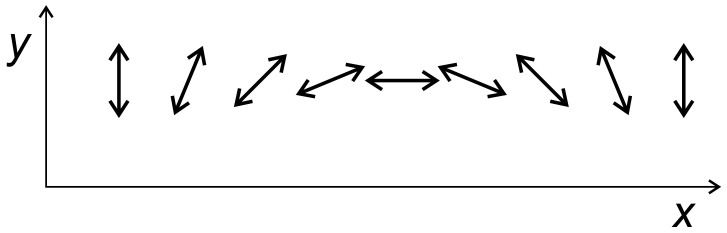
Scheme of continuous polarization rotation.

In earlier studies [34,38], we analyzed the light-induced directed motion of azo-polymer chains for modulated light intensity with constant polarization vector, corresponding to DTWM experiment with s−s or p−p linearly polarized beams. The current study casts some light onto the origin of polymer chain mass transport in the case of another DTWM experimental setup, with two circular polarized beams, the first left-handed, and the second right-handed [59]. The resulting illumination setup has constant intensity of light ($I\left(x,y\right)=I_{0}$), but the linear polarization vector rotates periodically along, e.g., the *x* axis (see Figure 9).

**Figure 10:**
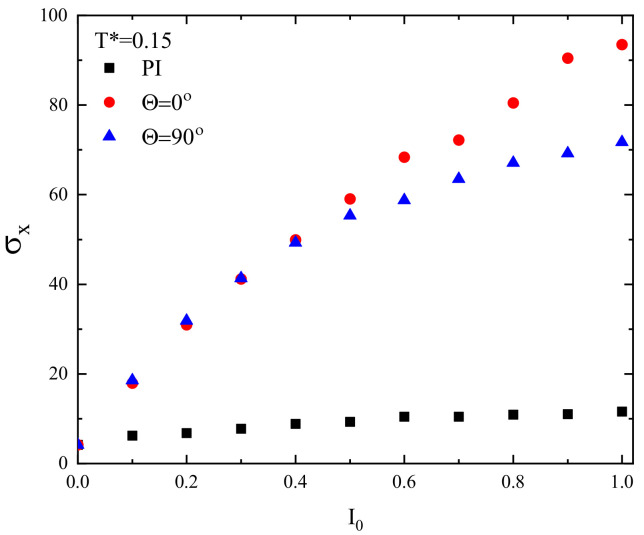
Plot of standard deviation $\sigma_{x}$, Equation (5), for FPM with linear polarizations $\Theta =0^{\circ }$ (red circles) and $90^{\circ }$ (blue triangles). Black squares, PI model.

The qualitative physical picture of the photoinduced dynamics is as follows. The driving force arises due to the slight asymmetry of the distribution of chains (Figure 6b,c) and of CM (Figure 7a,b) in the directions parallel and perpendicular to the polarization vector. This effect in, e.g., the *x* direction, can be characterized in terms of standard deviation of displacement $\sigma_{x}$ defined as:

(5) $$\sigma_{x}=\sqrt{{\left(\Delta {\vec{r}}^{\left(CM\right)}\right)}^{2}},$$

where in Equation (3) only the *x* component of displacement vector is taken into account: ${\vec{r}}_{i}^{\left(CM\right)}\left(t\right)=\left(x_{i}^{\left(CM\right)}\left(t\right),0\right)$. The plot of $\sigma_{x}$ in function of light intensity $I_{0}$ is shown for FPM and two polarization directions $\Theta =0^{\circ }$ and $\Theta =90^{\circ }$ in Figure 10. For sufficiently high light intensity ($I_{0}>0.5$), the mobility of chains along the *x* direction, represented by $\sigma_{x}$, becomes larger at the center of the illumination pattern, where $\Theta =0^{\circ }$, than at its in its end-points, where $\Theta =90^{\circ }$ (Figure 9). In both locations, the motion of the chains is symmetric with respect to left–right directions, but, because of different mobilities, an inhomogeneous distribution of the chains can build up. The mobility in PI model is at least one order of magnitude lower than in FP model.

This argumentation can be generalized by taking into account the modulation of mobility corresponding to continuous rotation of light polarization pattern shown in Figure 9. The resulting mass distribution will be more complex than for standard surface relief gratings—further studies are necessary to quantify this effect.

### 3.3. Macromolecular Displacement of Azo-Polymer under Linearly Polarized Light Illumination with Variable Intensity

The results presented in Figure 6b,c and Figure 7a,b show that constant linearly polarized light does not trigger off any directed mass transport of polymer chains. The same conclusion holds for the 3D case [34,38]. On the other hand, the directed movement in the simplified polarization model in 3D was promoted by the gradient of linearly polarized light intensity [34,38]; the mass transport took place from brighter to darker illumination areas.

In what follows, we analyze this aspect of chain dynamics benefitting from the simplicity of an analysis of polarization effects in 2D. We use the methodology developed in Ref. [38] and analyze the dynamics of the CM of the chains in linearly polarized light with an intensity gradient ($\nabla I=\frac{\partial I}{\partial x}$) along the *x* direction:

(6) $$I\left(x,y\right)=I\left(x\right)=I_{0}-\nabla I\;\left(x-x_{0}\right),$$

where $x_{0}$ denotes center of lattice in the *x* direction ($x_{0}=250$) and $I_{0}=I\left(x_{0}\right)$ is the intensity offset.

**Figure 11:**
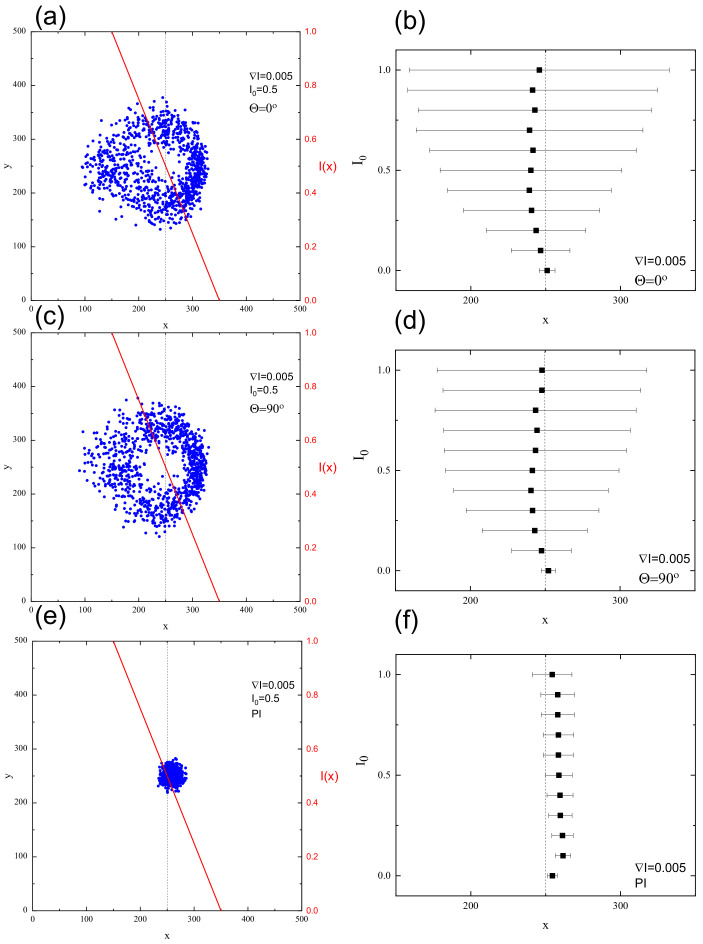
Final positions of CM of chains after $10^{4}$ MCS and averaged position in *x* direction with standard deviation $\sigma_{x}\left(I_{0}\right)$: for FPM with linear polarization with $\Theta =0^{\circ }$ (**a**,**b**) and $\Theta =90^{\circ }$ (**c**,**d**); and for PI model (**e**,**f**). $\nabla I=0.005,I_{0}=0.5$.

**Figure 12:**
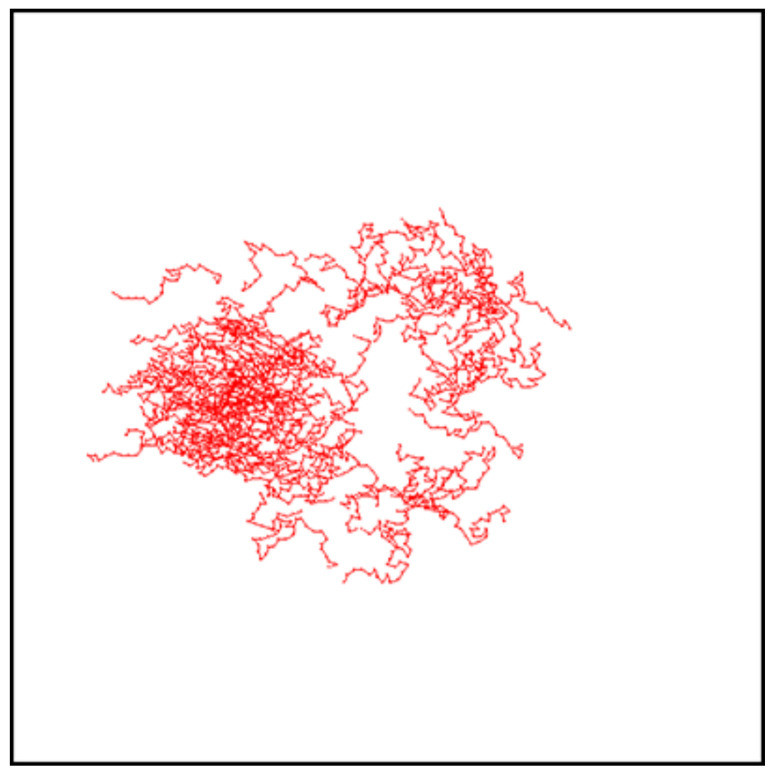
Instantaneous configurations of chains in FPM after 6000 MCS. $\nabla I=0.005,I_{0}=0.5$ and $\Theta =0^{\circ }$. One hundred chains are shown.

**Figure 13:**
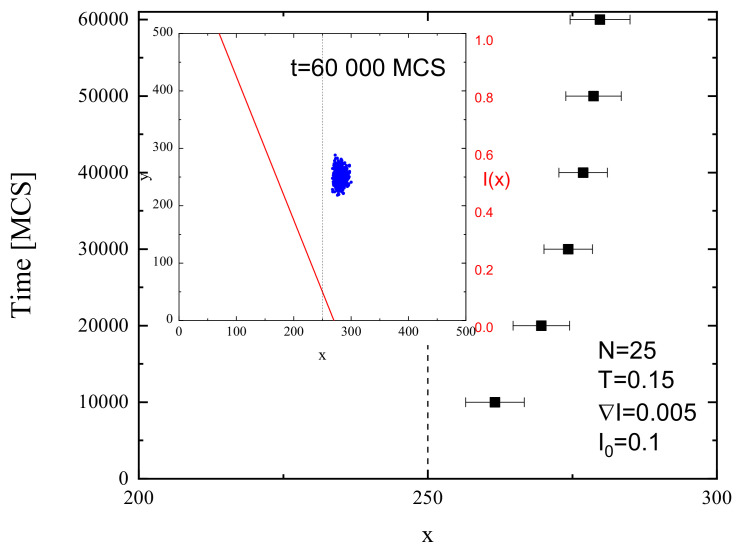
Averaged position of CM in *x* direction in function of MC time for PI model and final positions of CM of chains after $t=6\times 10^{4}$ MCS (inset). $\nabla I=0.005,I_{0}=0.1$

Figure 11 shows the final positions of CM of $10^{3}$ chains (left) and their averaged position along the *x* axis with standard deviation $\sigma_{x}\left(I_{0}\right)$ (right) after $10^{4}$ MCS for FPM with linear polarization $\Theta =0^{\circ }$ (Figure 11a,b) and $\Theta =90^{\circ }$ (Figure 11c,d) and for PI model (Figure 11e,f). The distribution of CM for FPM with linear polarization qualitatively resembles its counterpart for constant illumination (Figure 7); its visual asymmetry along the *x* axis results from variability of light intensity I(x). This asymmetry is also present in the distribution of the chains, shown after 6000 MCS in Figure 12. A rather unexpected effect is that the chains move away from their initial position in both directions—along increasing (left) or decreasing (right) illumination intensity. The chains which move to the left have higher mobility than those which move to the right. Despite this apparent asymmetry, there is no directed mass transport—the average position of the CM remains in initial position x=250. The PI model, on the contrary, shows a very different dynamics of CM of the chains—their spatial distribution is slightly shifted to the right. In this phase of the motion, some of the chains moved from darker to brighter areas. For a longer time interval ($6\cdot 10^{4}$ MCS, Figure 13 (inset)), all the chains moved from brighter to darker areas, generating a directed mass transport characterized in Figure 13 by the dependence of an average position of the chains in function of MC time. A similar effect was found in 3D for SPM [34,38]. Those topics are discussed in Section 4.

**Figure 14:**
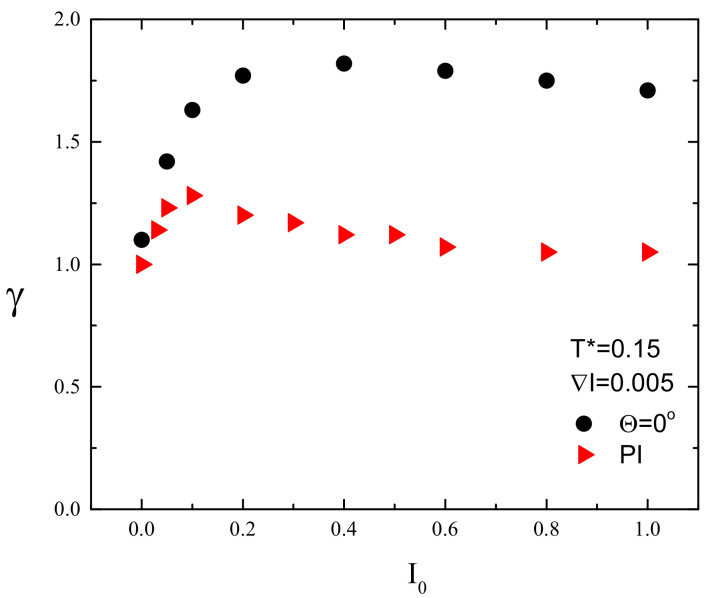
Exponent γ for light illumination with gradient ∇I=0.005. FPM with $\Theta =0^{\circ }$ (black circles) and PI model (red triangles). Experimental errors are of the size of graphical symbols.

The empty area in the center of distribution of CM (Figure 11a,b and Figure 12) resembles that in Figure 6b,c and Figure 7a,b and indicates the presence of superdiffusive dynamics. This conjecture is fully supported by Figure 14, which shows the plot of exponent γ as function of intensity offset $I_{0}$ for ∇I=0.005 and $\Theta =0^{\circ }$. This plot shows a strong resemblance to its counterpart for constant illumination (Figure 8). The PI model, on the contrary, displays moderate superdiffusion for lower values of offset $I_{0}$ and approaches diffusive dynamics for stronger illumination.

Finally, let us address the important topic of the reproducibility of the results. Monte Carlo simulations use random numbers to sample configurations and thus the bare data are not identical when two different sets of random numbers are used. In particular, the instantaneous configurations of the chains can be different. On the other hand, the average values of the parameters coincide within the experimental errors, if the system is not driven by large fluctuations (e.g., as in the case of critical state [57]). In our system, large fluctuations are absent and the results are reproducible in the above sense.

## 4. Discussion and Conclusions

We analyzed the impact of light polarization onto photoinduced dynamics of model azo-polymer chains using Monte Carlo simulations within the framework of bond-fluctuation model in two dimensions. The 2D case offers a much simpler technical analysis and visualization than its 3D counterpart. On the other hand, we argue that some of the results are generic and not related to reduced dimensionality of the polymer system.

A partial, rather technical discussion of the results accompanies the presentation of the results. Here, we discuss them in a wider context. Two main results of the paper cast more light onto the model photoinduced dynamics of azo-polymer chains.

The first result, supported by quantitative results, has a qualitative character—namely, the linear polarization of light plays an important role. Various schemes of the light–matter coupling in specific models can promote very different dynamics of photoinduced motion of azo-polymer chains. The two limiting cases were studied: (1) a polarization independent (PI) model, when all the azo-dyes undergo photoisomerization cycles; and (2) the model when those cycles become conditional upon the $\cos^{2}\theta$, thus accounting for all polarization effects. The PI model promotes a diffusive motion (γ=1), while in the second case a strong superdiffusive, nearly ballistic motion (γ=1.8) is present (Figure 8). The corresponding configurations of polymer chains and spatial distributions of their centers of mass (CM) (Figure 6 and Figure 7) are qualitatively different, expressing various types of underlying dynamics. In addition, the FPM promotes a partial orientation of the chains along the polarization direction, while the PI model leaves the chains disoriented (Figure 6).

The two limiting models for polarization effects result in a very different overall mass transport in the case of inhomogeneous linearly polarized illumination. The PI model promotes a directed mass transport, in the direction of decreasing illumination. On the contrary, the FPM is devoid of this attribute (Figure 11). As in the case of constant illumination, for sufficiently high values of offset illumination, in the former case, the dynamics is diffusive, while in the latter it is strongly superdiffusive (Figure 14).

The second result is the absence of directed mass motion for FPM in the case of inhomogeneous linearly polarized light (Figure 11). While the superdiffusive dynamics of chains along and against the gradient of illumination are different (Figure 11 and Figure 12), an overall mass transport does not exist. Preliminary simulations show that the direction of motion of a chain depends on its initial conformation.

Those results, rather arbitrarily extrapolated onto the 3D case, indicate that the SPM can be oversimplified and a more sensitive to polarization light–matter coupling may lead to new effects, in particular when applied to SRG inscription or to more complex polarization patterns as, for example, the continuous rotation model introduced in Section 3.2.2.

As in the 3D case, the dynamics of single chains is, in most cases, superdiffusive. This feature seems to be generic and supports the conclusion [38] that models of light-driven transport of azo-materials based on normal diffusion are inadequate and more advanced methods of modeling of anomalous diffusion (continuous time random walk and stochastic differential equations) should be used. Moreover, this study casts some light onto the origin of superdiffusive dynamics in model physical systems having close relation to experimental studies. Namely, the PI model displays a symmetry in the sense that the azo-dyes are interacting with light in the same way, independent on the light polarization. This symmetry leads to standard diffusion. The FPM fully breaks this symmetry and promotes strongly superdiffusive (nearly ballistic) dynamics. Thus, it is plausible that a weak breaking of the symmetry results in a transition form standard diffusion to weak superdiffusion, with value of γ slightly above 1. This conjecture is partially supported by the 3D case, when the symmetry is slightly broken, as discussed in Section 1, and γ≈1.25 [38].

The dynamics of an azo-polymer chain results from a complex interplay of motions of the monomers due to their thermal motions as well as photoisomerization events. The decomposition of the resulting dynamics onto those two components constitutes a challenge. On the other hand, some preliminary conclusions can be drawn. Namely, the pure photoisomerization dynamics results in a nearly perfect orientation of a chain along the polarization direction without translation of the chain as a whole (Figure 6a). When this dynamics is accompanied by thermally-driven dynamics, a strong superdiffusive translation appears (Figure 6b,c) with partial orientation of the chains. However, the thermally-driven dynamics is not sufficient to promote strong translation (see Figure 6d). In this case, the photoisomerization is independent of the polarization (PI model) and the translation dynamics is diffusive.

The conclusions drawn from current simulations have, in some cases, a qualitative or semi-quantitative character. Their systematic analysis requires further studies which go beyond the scope of this paper and will be subject of forthcoming publications.
